# Night Air

**Published:** 1880-10

**Authors:** 


### NIGHT-AIR.

MUCH that is untrue has been communicated to the public as to the healthfulness and unhealthfulness of night-air, for want of enlarged information and opportunities of observation on the part of the writers. There is no one universal, safe rule; what is a healthful practice in one latitude, or one locality, or one season, may be deadly in another. Peculiarity of constitution modifies all rules on this subject. The only safe plan, therefore, is, to enunciate certain well-established principles, which the intelligent reader must wisely apply in practice.

There is nothing necessarily injurious in the night-air, even to feeble, delicate persons, in our latitude, or any season of the year, provided a hot meal has been taken, and the person exercises with sufficient activity to be comfortably warm, and keep off a feeling of fatigue; but as soon as the exercise ceases, shelter should be taken in a house. With these precautions, exposure to the night-air is a positive good to sick and well, as a very general rule, because the in-door air is the out-door air mixed with various in-door sources of impurity.

Except in the miasmatic season of the year, which, in latitudes north of thirty-five, may be embraced between the first days of August and October, most persons may sleep with impunity, and even with advantage, in the open air, with the sky for a canopy, if there is enough covering to keep the body comfortably warm. Whether in sleeping in a house, the windows should be open or closed, depends on the part of the house occupied, and on the season of the year. Many a life is annually lost by not taking these points into account. In our book on "SLEEP," the laws of miasm are clearly stated; and this can not be done too often, nor can they be too well or too generally understood, because it is a subject of vital importance to every human being.

When it is cool enough to keep fires in the house all day, and until the nights begin to get cool in the autumn, it is far better for both sick and well, whether sleeping in the cellar or in the garret, to have an abundant supply of out-door air coming into the chamber from windows let down at the top, and raised from the bottom, more or less, according to the size of the room, the number occupying it, and the season of the year; always, however, arranging that a draught of air should not be on the sleeper.

In the miasmatic season, a discrimination should be made, and a sound judgment should be exercised. The general rule is, that during August and September, when the days are hot and the nights are cool, in flat localities, along watercourses, and near lakes and ponds, persons sleeping on the ground-floor should close all the outer doors and windows, but in the upper stories the windows may be opened, and the inner doors closed.

because the cold keeps the miasm, the disease-engendering agency, near the earth's surface, within the first five or six feet, and lower in proportion to the greater coolness; as you ascend above that point, the air becomes purer and purer. Some of this malignant air will enter the chamber through the crevices of the windows and doors, although they may be closed; but it enters in such small quantities that it is immediately warmed, for it is ordinarily ten degrees warmer in-doors than without, at night, in the autumn; and this greater warmth rarefies the incoming air, and sends it at once to the ceiling, where it can not be breathed. As this bad air is below the upper stories of a building, the windows may be opened; yet, as inside the building, it seeks the upper portions, the inner doors should be closed, so that it should be admitted in as small quantities as possible. The great general fact is this: miasm and carbonic acid gas, mingled in the air we breathe, will cause death in a month, or in a minute, according to their concentration, according to the strength of the mixture. Cold causes them both to seek the surface of the earth or the floor; hence in a cold chamber the nearer the ceiling you sleep the purer the air is; but in a warm chamber the purest air is on the floor, as the warmth sends the poisonous gases to the ceiling. At the first glance all this appears vexatiously complex; but when the natural law on the subject is clearly perceived, it is beautifully simple, as all God's laws are, and as wise and beneficent as they are beautiful and clear

*BEAUTY UNDER A CLOUD*

May be relieved from any blemish caused by ordinary eruptions, such as pimples, dry exfoliations, cold sores, scurf, or what is called muddiness of the complexion, by a course of STAFFORD'S IRON AND SULPHUR POWDERS. They act upon these disfigurements and discolorations in the venous blood—their operation being exactly reverse of the suppressive washes and lotions, all of which are more or less dangerous. Instead of driving the impurities back into the system, to reappear in other parts of the body and in other and more virulent forms, the Powders cause them to be exhaled through the pores.

Sold by Druggists. 1 Package, 12 Powders, $1; 6 Packages, 72 Powders, $5. Mailed free. HALL & RUCKLE, 218 Greenwich Street, New York.

